# Ingestion of green tea with lowered caffeine improves sleep quality of the elderly via suppression of stress

**DOI:** 10.3164/jcbn.17-6

**Published:** 2017-09-05

**Authors:** Keiko Unno, Shigenori Noda, Yohei Kawasaki, Hiroshi Yamada, Akio Morita, Kazuaki Iguchi, Yoriyuki Nakamura

**Affiliations:** 1 Department of Neurophysiology, School of Pharmaceutical Sciences, University of Shizuoka, 52-1 Yada, Suruga-ku, Shizuoka 422-8526, Japan; 2 Tea Science Center, Graduate Division of Nutritional and Environmental Sciences, University of Shizuoka, 52-1 Yada, Suruga-ku, Shizuoka 422-8526, Japan; 3 Division of Drug Evaluation & Informatics, School of Pharmaceutical Sciences, University of Shizuoka, 52-1 Yada, Suruga-ku, Shizuoka 422-8526, Japan; 4 Department of Functional Plant Physiology, Faculty of Agriculture, Shizuoka University, 836 Ohya, Suruga-ku, Shizuoka 422-8529, Japan

**Keywords:** elderly people, sleep, green tea, theanine, salivary α-amylase

## Abstract

Epidemiological and animal studies have demonstrated that ingestion of green tea enhances healthy life. However, caffeine in green tea can interfere with sleep. In this report, we examined the effect of green tea with lowered caffeine, low-caffeine green tea, on stress and sleep of the elderly. The participants (*n* = 10, mean age 89.3 ± 4.2 years) drank five cups/day of standard green tea for 1 week. Subsequently, they drank five cups/day of low-caffeine green tea for 2 weeks. Salivary α-amylase activity (sAA) was measured as a stress marker. Sleep stages were measured using a portable electroencephalography (*n* = 7, 6 female and 1 male). The level of sAA in the morning (sAAm) was significantly lower when the participants drank low-caffeine green tea than standard green tea. While the levels of sAAm were different among individuals, lower sAAm correlated with a higher quality of sleep. In those participants whose sAAm was lowered by the ingestion of low-caffeine green tea, some sleep parameters improved. Daily ingestion of low-caffeine green tea may be a beneficial tool for improving the quality of sleep of the elderly via the suppression of stress, although further research is required to fortify this hypothesis.

## Introduction

Green tea is the most popular drink in Japan and Asian countries. Epidemiological data shows that daily ingestion of green tea lowers the risk of dementia.^(1–3)^ However, some elderly people refrain from drinking green tea because they know that caffeine in green tea disturbs their sleep, suggesting that if green tea does not interfere with sleep, then it would be especially beneficial for the elderly. We then examined whether the ingestion of green tea with lowered caffeine content was able to improve the quality of sleep of the elderly that usually ingested caffeine derived from green tea. Caffeine has profound effects on sleep and wake function.^(4)^ Laboratory and epidemiological studies have documented the sleep-disruptive effects of caffeine such as an increase in sleep onset latency, a decrease in total sleep time and an adverse effect on sleep quality.^(5)^ Results related to the disturbance of caffeine on sleep have mostly been conducted on young to middle aged adults, where it has been shown that sleep quality generally decreases with aging.^(5,6)^

Green tea is mainly composed of catechins, caffeine, and amino acids. In rats, caffeine-induced sleep disturbances were partially counteracted by theanine.^(7)^ Theanine (l-theanine, *N*-ethyl-l-glutamine) is the major amino acid in tea leaves (*Camellia sinensis* L.), and has significant anti-stress effects on animals and humans.^(8–12)^ In tea leaves, other amino acids such as arginine (Arg), glutamic acid (Glu) and glutamine (Gln) are also contained. We recently noted that Arg has a significant anti-stress effect, similar to theanine, while Glu and Gln have no anti-stress effect.^(13)^ We also found that theanine [1/5 (w/w) of caffeine] and Arg [1/10 (w/w) of caffeine] cooperatively abolished the effect of caffeine on the adrenal hypertrophy of psychosocially stressed mice.^(13)^ On the other hand, catechins, mainly epigallocatechin gallate (EGCG), have potent antioxidative and anti-inflammatory activities that fortify the beneficial effect of green tea on health.^(14–16)^ However, EGCG potently suppressed the anti-stress effect of theanine, while epigallocatechin (EGC), the second most abundant gallate-free catechin, retained the effect of theanine.^(13)^ These results suggest that balances among theanine, caffeine, catechins and Arg are crucial for the function of green tea. Since the concentrations of caffeine, catechins and amino acids eluted into water are altered by the kind of tea leaves and water temperature, their content in each green tea solution needs to be measured.

We prepared green tea with lowered caffeine, and termed it low-caffeine green tea.^(13)^ Also, to reduce EGCG, we applied the temperature-sensitive kinetics of water elution on each tea component. Gallate catechins and caffeine were easily eluted in boiling water but not at room temperature, whereas the elution of theanine was almost unaffected by temperature.^(17,18)^ Indeed, the ingestion of low-caffeine green tea that was steeped in room temperature water significantly suppressed the stress response in mice.^(13)^

In this study, we examined the effect of low-caffeine green tea on sleep and stress of elderly people. Low sleep efficiency is associated with stress responses such as the level of salivary α-amylase activity (sAA) and cortisol in the morning.^(19,20)^ The activity of sAA increases rapidly in response to physiological and psychosocial stress.^(21–23)^ High-level sAA in the morning has been observed in children with poor sleep.^(20)^ Therefore, a decrease of sAA in the morning (sAAm) may serve as a marker for improved sleep quality. Sleep stages were measured using a portable electro-encephalography. Some parameters pertaining to sleep and sAAm changed after the ingestion of low-caffeine green tea. In addition, the associations between sleep, cortisol regulation and diet are reportedly implicated with the risk of dementia.^(24)^ The disturbance of sleep is strongly associated with behavioral and psychological symptoms of dementia at a very early stage of Alzheimer disease.^(25)^ The intervention on sleep quality with dietary supplements may be a potential therapeutic strategy for a healthier life, especially in the elderly.

## Materials and Methods

### Preparation of low-caffeine green tea

Fresh tea leaves were treated with a hot water shower at 95°C for 180 s to elute out caffeine from tea leaves as described previously.^(13)^ Then, the tea leaves containing a lowered caffeine content were dried by a standard manufacturing process. We termed this low-caffeine green tea.

Since the low-caffeine green tea was eluted with room temperature water, twice the amount of tea leaves of low-caffeine green tea than standard green tea was used (Table 1). Tea bags of low-caffeine green tea (total 20 g) were steeped in 2 L of room temperature water for 5 min, and then tea bags were well stirred. The tea bags were removed 10 min later and the eluate was warmed to 60–70°C. Standard green tea (total 10 g) was steeped in 2 L of boiling water. Fresh tea was prepared five times a day by the care center staff.

### Measurement of tea components by high performance liquid chromatography (HPLC)

The eluates of low-caffeine and standard green teas described above were measured by HPLC as described previously.^(13)^ In brief, catechins and caffeine in the eluates were measured by HPLC (SCL-10Avp, Shimadzu, Japan; Develosil packed column ODS-HG-5, 150 × 4.6 mm, Nomura Chemical Co., Ltd., Japan) according to the method of Horie *et al.*^(26)^ Catechins and caffeine were measured at 280 nm. Free amino acids in tea leaves were measured by HPLC as described above using homoserine as an internal standard.^(27)^ Amino acids were detected at an excitation wavelength of 340 nm and at 450 nm of emission wavelength (RF-535 UV detector, Shimadzu, Japan). The relative standard deviation (RSD%) of precision and repeatability were <5.0%. The recoveries of catechins, caffeine, and free amino acids were 99 ± 4%, 98 ± 4%, and 98 ± 3%, respectively.

### Participants

Ten elderly people (*n* = 10, 9 female and 1 male, mean and SD 89.3 ± 4.2 years), who lived in a nursing home, received verbal and written information about the study and signed an informed consent form before entering the study. None of the participants indicated acute or chronic disease, regular intake of medication, or habitual smoking. Although they drank green tea every day, they did not show symptoms of subjective insomnia due to the intake of green tea. Participants were instructed to drink mainly the test tea, and not to take theanine- and caffeine-rich beverages such as other teas, including green tea, coffee, black tea and soda throughout the experiment. In addition, they did not also consume caffeine-rich foods such as chocolate and candy. Since the participants were drinking green tea every day at each meal and tea time, the ingestion of green tea was considered to be a baseline condition for each participant. In addition, five cups of green tea was a normal amount for these participants. The study was conducted in accordance with the Declaration of Helsinki and Ethical Guidelines for Medical and Health Research Involving Human Subjects (Public Notice of the Ministry of Education, Culture, Sports, Science and Technology and the Ministry of Health, Labour and Welfare, 2015). This study was approved in Japan, and the study protocol was approved by the Ethics Committee of the University of Shizuoka (No. 26–41). This study was registered at the University Hospital Medical Information Network (UMIN) (registration ID no. UMIN16533). The study period was from February to March, 2015.

### Procedure

This study was a single arm and non-randomized design. The participants drank standard green tea for 1 week and subsequently drank low-caffeine green tea for 2 weeks at breakfast, lunch, dinner, and morning and afternoon tea times. Sleeping hours and the sAA of each participant was recorded in a questionnaire by the care center staff every day for 3 weeks. Subjective stress in the evening was evaluated using visual analog scales (VAS: 0–10) from very relaxed to highly stressed and recorded in a questionnaire by the care center staff every day for 3 weeks. An overnight electroencephalogram (EEG) was monitored for 6 days, 3 days (from Wednesday to Friday) in the period of standard green tea, and 3 days in the last week of low-caffeine green tea. When EEG measurements were made for all 3 days, data from the second day in the period of the measurement was used for analysis.

### Measurement of sAA

To assess the physiological stress response, sAA was measured using a testing strip and a colorimetric system (Nipro Co., Osaka, Japan).^(28)^ Briefly, the testing strip consists of a collecting strip used to collect saliva and a reagent strip used to measure sAA. A substrate 2-chloro-4-nitrophenyl-4-*O*-β-d-galactopyranosylmaltoside in the reagent strip is hydrolyzed by salivary amylase in the presence of maltose, a competitive inhibitor. This reaction turns the color of a reagent strip from white to yellow, and the change is quantified using a colorimetric system (salivary amylase monitor). One unit of activity (U) per mass of enzyme is defined as the production of 1 µmol of the reducing sugar, maltose, in 1 min (NC-IUBMB, 1992). Saliva was measured twice a day, the first time prior to breakfast and within 1 h after waking up, and the second time before sleep, but after dinner. This was done for 3 weeks throughout the practice. Prior to sampling, participants washed their mouths with water. After saliva was collected for 30 s using a testing strip, care staff measured the participants’ sAA.

### Measurement of EEG

EEG monitoring was achieved using a single-channel EEG (Sleep Scope, SleepWell Co., Osaka, Japan),^(29,30)^ as previously described for sleep scoring programs.^(31,32)^ The channel located at approximately Fpz-M1 was recorded for the 1ch EEG system. Before sleep, two electrodes of the Sleep Scope were attached to the participant’s forehead and mastoid to collect electrophysiological signals by a care staff. Measurements started when participants entered their bed. After participants woke up and exited their bed, measurements were stopped.

The data was routinely analyzed at the SleepWell Co. (Osaka, Japan) and categorized into rapid eye movement (REM) sleep and non-REM sleep, which was again classified into light sleep (N1/N2) or slow-wave sleep (N3). The onset of sleep (SL) was defined as 5 min of continuous sleep. Total sleep time (TST) was calculated as the total period of sleep (SPT) minus the time spent awake during the sleep period (Total WASO). Sleep efficiency (SE) was the ratio of TST to the time in bed (TIB). Early morning awakening (B2 WASO) was evaluated from the time spent awake during two hours before the final awakening.

### Statistical analysis

All results are expressed as the mean ± SEM. Differences in sAA were evaluated using one-way analysis of variance (ANOVA) followed by a Tukey-Kramer post hoc test for multiple comparisons. A paired *t* test was carried out between sAAm and sleep parameters, and on differences between sAAm and several sleep parameters from standard green tea intake to low caffeine green tea intake. All statistical analyses were carried out using a statistical analysis system (SAS 9.4, SAS Institute Inc., Cary, NC). In each analysis, a *p* value <0.05 was considered to be statistically significant.

## Results

### Tea components in standard and low-caffeine green teas

The content of caffeine in low-caffeine green tea steeped in room temperature water was about 1/3 that of standard green tea steeped in boiling water (Table 1). Although EGCG was the main catechin in the eluate of standard green tea, EGC was the most abundant catechin in the eluate of low-caffeine green tea. Other gallate-type catechins, epicatechin gallate (ECG) and catechin gallate (CG), were lower in the eluate of low-caffeine green tea than in standard green tea. The total amount of catechins was not different between standard and low-caffeine green teas.

The amount of theanine in low-caffeine green tea was about 2.4 times higher than that in standard green tea. Arg in low-caffeine green tea was about 3 times higher than that in standard green tea. Other amino acids in low-caffeine green tea were about 2 times higher than those in standard green tea. The total amount of amino acids was 2.3 times higher in low-caffeine green tea than in standard green tea.

The participants drank five cups (150 ml × 5 cups = 0.75 L) of green tea each day. The ingestion of theanine was about 27 mg/day in standard green tea, and 64 mg/day in low-caffeine green tea. On the other hand, the ingestion of caffeine decreased from about 90 (120 × 0.75 = 90) mg/day to 27 (37.2 × 0.75 = 27) mg/day in standard and low-caffeine teas, respectively.

### Lowered sAAm by the ingestion of low-caffeine green tea

**Fig. 1:**
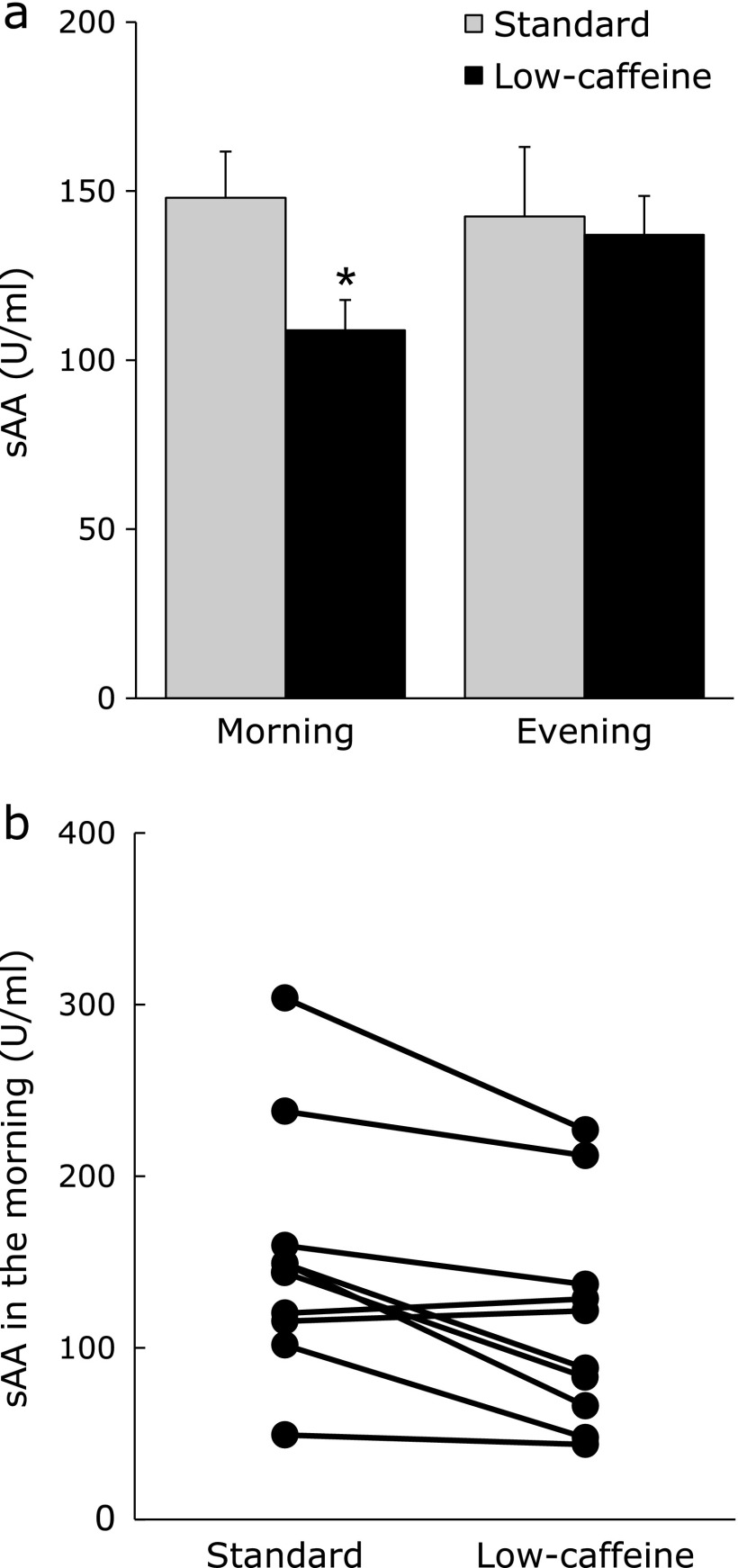
Salivary α-amylase activity (sAA) of the participants was measured in the morning after waking up and in the evening. Each participant drank five cups/day of standard (white column) and low-caffeine (black column) green teas. The mean sAA value of all participants (a), and mean value of each participant (b). Data are expressed as mean ± SEM (a) and mean (b) (*n* = 10, *p<0.05; one-way ANOVA).

The level of sAAm was significantly lower when the participants ingested low-caffeine green tea than standard green tea (Fig. 1a, *p* = 0.008). On the other hand, the level of sAA in the evening did not change after the ingestion of low-caffeine green tea. The change of sAAm for each participant is shown in Fig. 1b. Although there were individual differences in sAAm, eight of the participants showed lower sAAm when they drank low-caffeine green tea rather than standard green tea. The subjective stress that was checked in the evening was not changed by the ingestion of low-caffeine green tea (standard 4.19 ± 0.25; low-caffeine 4.68 ± 0.13).

### Effect of low-caffeine green tea ingestion on sleep parameters

The sleep data of seven participants (6 females and 1 male, mean and SD 89.6 ± 4.9 years) were obtained. One participant unconsciously removed the electrodes during measurements. Another participant refused to receive measurements. And yet another participant could not continue measurements caused by itchiness at the site where the electrode was attached.

**Fig. 2:**
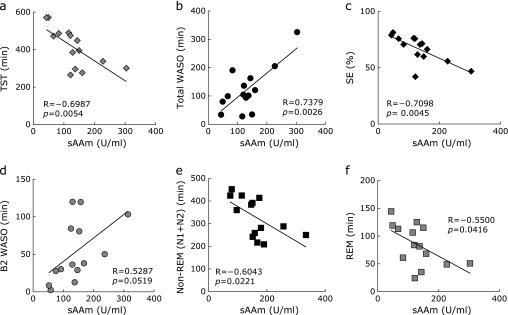
Correlation between sAAm and each sleep parameter. TST (a), total WASO (b), SE (c), B2 WASO (d), Non-REM (N1 + N2) (e), and REM (f).

Since there was no difference in sleep time of each participant in the period of the EEG measurements, data from the second day was used for the analysis. The data of sAAm and sleep parameters when each participant drank standard or low-caffeine green tea are shown in Table 2. Mean values of TIB, SL, SPT, TST, Total WASO, SE, Non-REM and REM did not change significantly after the ingestion of green tea from standard green tea to low-caffeine tea, even though B2 WASO tended to be shorter when participants ingested low-caffeine green tea than standard green tea (Table 2). Since the levels of sAAm were very different among individuals (Fig. 1b), the correlations between sAAm and sleep parameters were examined. The results showed that sAAm level was negatively correlated with TST and SE (Fig. 2a and c). Although SL was not correlated with sAAm (R = −0.3450, *p* = 0.227), the nocturnal awakening (total WASO) was significantly lowered in those participants with lower sAAm (Fig. 2b). In addition, REM and non-REM (N1 + N2) sleep were longer in those participants with lower sAAm (Fig. 2e and f).

**Fig. 3:**
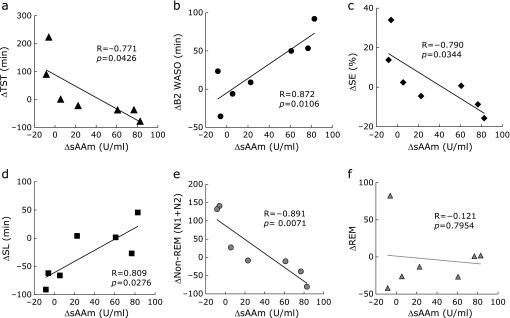
Differences of sAAm between standard green tea intake and low-caffeine green tea intake (ΔsAAm) were compared with differences in each sleep parameter between standard green tea intake and low-caffeine green tea intake. Correlations between ΔsAAm and ΔTST (a), ΔB2 WASO (b), ΔSE (c), ΔSL (d), ΔNon-REM (N1 + N2) (e), and ΔREM (f).

Next, in the participant whose sAAm was lowered by the ingestion of low-caffeine green tea, we examined whether sleep parameters had changed indeed. The difference of sAAm between standard green tea intake and low-caffeine green tea intake [ΔsAAm = sAAm (standard) − sAAm (low-caffeine)] was compared with the differences of each sleep parameter between standard green tea intake and low-caffeine green tea intake (Fig. 3). ΔsAAm was positively correlated with ΔB2 WASO and ΔSL, and negatively correlated with ΔTST, ΔSE and Δnon-REM (N1 + N2). These data indicate that when the change in sAAm was caused by the ingestion of low-caffeine green tea, there was some changes in sleep variables relative to each individual’s basal level.

## Discussion

### Effect of low-caffeine green tea ingestion on sAAm and sleep

This study is the first study to explore the effect of low-caffeine green tea on sleep quality of the elderly but has several limitations. Firstly, this study has a very small participant size and takes place under single arm non-randomized conditions. It may be difficult in a single-arm trial to interpret the effects of low-caffeine green tea without placebo effects. However, placebo effects on the data of sAA and EEG obtained in this study may be low because the data is not subjective. Secondly, the participants are considerably aged. Thirdly, since the participants were drinking green tea daily, their basal values of sAAm and sleep parameters were considered to be the values when they drank green tea. Therefore, our data may not be relevant to compare with other population groups. Fourthly, we used a single-channel EEG instead of gold-standard polysomnography (PLG) for sleep assessment. Stereoscopic information was not obtained using a single-channel EEG. However, it has recently been showed that single-channel EEG can be a useful research tool relative to single-channel EEG with PLG.^(33)^ Therefore, our data using single-channel EEG is considered to provide comparable results to PLG in assessing REM, non-REM sleep and several other parameters.

Whereas the level of sAAm is usually low at the time of waking up but becomes high as a result of sympathetic excitement during the day,^(34)^ if stress is small, then no significant change is observed. The participants in this study were considered to have little stress during the test period. When the period of sleep is short, sAAm becomes high,^(20)^ and ingestion of caffeine increases sAA.^(35)^ In this study, the lower sAAm was positively correlated with the shorter nocturnal awakening and longer non-REM sleep, indicating that the level of sAAm is closely correlated with sleep length and quality. While sAAm is different among individuals, the change of ingestion from standard to low-caffeine green tea lowered the level of sAAm in eight among ten participants. Despite several limitations, in those participants with a large decrease in sAAm, some sleep parameters improved. Therefore, the ingestion of low-caffeine green tea is considered to be a beneficial tool for improving the sleep quality of the elderly via the suppression of stress.

Sleeping abnormalities are associated with an increase in cognitive decline,^(24,25,36)^ and thus it is important to identify those factors that contribute to difficulties in sleeping. The total amount of caffeine is negatively correlated with the number of times individuals get up at night and with aberrant motor behavior in elderly participants with dementia.^(37)^ Decreased ingestion of caffeine may benefit the quality of sleep of elderly, even if they have no subjective insomnia at that time.

### Relationship between sleep and tea components

A number of studies have indicated that regular daily dietary caffeine intake is associated with disturbed sleep.^(5)^ Caffeine-induced wakefulness is determined by caffeine consumption, the rate of caffeine metabolism and advanced age.^(5,38,39)^ However, the decreased caffeine in low-caffeine green tea may not be the only reason for the altered sleep of the elderly. The effect of caffeine on sleep was suppressed by theanine.^(7)^ In addition, the effect of caffeine on stress response was suppressed by theanine and other tea components such as Arg and catechins.^(13)^ The stress response is thought to closely correlate with sleep via the sympathetic–adrenal–medullary axis and the hypothalamic–pituitary–adrenal axis.^(19,20)^ Therefore, the anti-stress effect of theanine and other tea components may be important for sleep quality. In the low-caffeine green tea eluted in room-temperature water, the caffeine content was 0.4-fold that of theanine (Table 1), suggesting that the effects of caffeine were almost completely abolished by the cooperative effect of theanine and Arg.^(13)^ Whereas EGCG also has a potent counter-effect against theanine, Arg (1-fold of EGCG) or EGC (10-fold of EGCG) cooperatively abolished the effect of EGCG with theanine (2-fold of EGCG).^(13)^ This suggests that the effect of EGCG in the low-caffeine green tea was also abolished. On the other hand, in standard tea, the contents of caffeine and EGCG were both 3.3-fold that of theanine, suggesting that theanine could not suppress the effect of caffeine and EGCG, even though assisted by Arg and EGC.

Theanine has been reported to increase sleep quality in normal volunteers,^(40)^ in patients with schizophrenia,^(41)^ in boys with attention deficit hyperactivity disorder.^(42)^ In addition, the anti-stress effect of theanine has been observed in healthy adults using magnetoencephalography.^(12)^ However, the underlying mechanism is not known. The role of GABA in sleep induction and maintenance is well accepted.^(43,44)^ In the hippocampus of mice that ingested theanine for 2 weeks, the level of GABA increased, and conversely the levels of glutamate (Glu) and pyroglutamic acid were significantly reduced.^(45)^ Glu serves as the precursor for the synthesis of GABA in GABA-ergic neurons by glutamate decarboxylase. The mechanism by which the level of GABA is increased by theanine has not been elucidated. Since Glu is the main excitatory neurotransmitter and GABA is the chief inhibitory neurotransmitter in the mammalian central nervous system,^(46)^ theanine modulates a balance between GABA and Glu. On the other hand, lack of sleep quality has been reported to increase oxidative stress in the central nervous system.^(47)^ Theanine may have another role in sleep by providing neuroprotection against oxidative stress-induced neuronal damage.^(48)^

Decreased EGCG and increased EGC in low-caffeine green tea may modulate the effect of GABA because EGCG has been reported to modulate the GABA_A_ receptor.^(49)^ In addition, Arg is considered to be an important regulator in the central nervous system through the synthesis of nitric oxide.^(50)^ An increase in nitric oxide production in the basal forebrain is a causal event in the induction of recovery sleep.^(51)^ While Arg is highly included in several foods such as soybean, meat and fish, further studies are needed to assess whether both theanine and Arg are important for the improvement of sleep quality.

In previous studies,^(40–42)^ purified theanine (200–400 mg/day) was used to improve sleep. Our study suggests that a lower dose of theanine is effective under the cooperative effect of Arg and EGC. An additional large-scale clinical trial would be required to determine the effect of low-caffeine green tea. In addition, since each baseline level of sleep and stress sensitivity is different among individuals, a detailed analysis that is based on individual differences is needed.

## Conclusions

We examined whether the ingestion of low-caffeine green tea is able to suppress the stress response and improve the quality of sleep in the elderly. The contents of theanine, Arg and EGC were higher while caffeine and EGCG were lower in low-caffeine green tea than in standard green tea. Although our study has several limitations such as a very small size (sAA data, *n* = 10, 9 females and 1 male; EEG data, *n* = 7, 6 females and 1 male) and a very old population (mean age 89.3 ± 4.2 y), there was a significant correlation between sAAm and sleep parameters. Lower sAAm was closely correlated with higher sleep quality. In those participants whose sAAm was lowered by the ingestion of low-caffeine green tea, some sleep parameters improved. Thus, daily ingestion of low-caffeine green tea that has been steeped in room temperature water may be a tool for improving the quality of sleep of the elderly via the suppression of stress, although further research is required to fully assess the effect of low-caffeine green tea.

## Figures and Tables

**Table 1:** The content of caffeine, catechins and amino acids in a solution of low-caffeine green tea

Green tea	Caffeine (mg/L)	Catechins (mg/L)						
		EGCG	EGC	ECG	EC	CG	(+) C	Total
Standard	120 ± 6.75	117 ± 0.58	73.2 ± 2.84	26.5 ± 1.78	43.9 ± 5.33	0.41 ± 0.046	5.47 ± 0.81	266 ± 9.85
Low-caffeine	37.2 ± 1.35	64.7 ± 2.6	145 ± 4.52	10.7 ± 0.2	53.8 ± 4.17	0.26 ± 0.05	4.13 ± 0.32	278 ± 9.01

Green tea	Free amino acids (mg/L)									
	Theanine	Glu	Arg	Asp	Gln	Ser	Ala	Asn	GABA	Total
Standard	36 ± 2.65	9.12 ± 0.81	7.28 ± 0.41	7.44 ± 0.5	5.34 ± 0.44	2.99 ± 0.09	0.90 ± 0.03	0.76 ± 0.03	0.47 ± 0.03	70.4 ± 4.9
Low-caffeine	85.2 ± 5.45	18.2 ± 1.39	21.6 ± 1.69	12.9 ± 0.76	15.8 ± 1.01	5.73 ± 0.41	2.16 ± 0.15	1.21 ± 0.1	1.51 ± 0.18	164 ± 11.1

Low-caffeine green tea (20 g) was steeped in 2,000 ml of room temperature water. Standard green tea (10 g) was steeped in 2,000 ml of boiling water. EGCG, (–) epigallocatechin gallate; EGC, (–) epigallocatechin; ECG, (–) epicatechin gallate; EC, (–) epicatechin; CG, (–) catechin gallate; (+) C, (+) catechin; Glu, glutamic acid; Arg, arginine; Asp, aspartic acid; Gln, glutamine; Ser, serine; Ala, alanine; Asn, asparagine; GABA, γ-amino butyric acid.

**Table 2:** Sleep parameters and sAAm in each participant

No	AgeSex	Green tea	sAAm (U/ml)	TIB (min)	SL (min)	SPT (min)	TST (min)	Total WASO (min)	SE (%)	B2 WASO (min)	Non-REM (min)			REM (min)
											N1	N2	N3	
1	92	Standard	143.7 ± 40.0	628.0	11.5	611.0	448.5	162.5	71.4	80.5	190.0	223.5	0.0	35.0
	F	Low-caffeine	82.8 ± 18.4	686.5	10.0	676.0	485.5	190.5	70.7	30.5	124.5	300.0	0.0	61.0

2	94	Standard	49.1 ± 17.6	700.0	48.0	651.0	570.5	80.5	81.5	2.5	25.0	426.5	0.5	118.5
	F	Low-caffeine	43.7 ± 21.3	719.0	114.0	603.5	569.0	34.5	79.1	8.5	68.5	356.0	0.0	144.5

3	89	Standard	303.7 ± 34.2	640.5	9.0	627.0	301.0	326.0	47.0	103.5	186.0	64.0	0.0	51.0
	M	Low-caffeine	226.9 ± 43.2	605.5	36.0	543.0	337.5	205.5	55.7	50.0	42.0	246.0	0.0	49.5

4	84	Standard	149.0 ± 28.8	656.5	95.0	430.5	395.5	35.0	60.2	120.0	30.0	250.5	0.0	115.0
	F	Low-caffeine	66.2 ± 15.7	623.5	49.5	573.0	473.0	100.0	75.9	28.0	106.5	253.5	0.0	113.0

5	86	Standard	159.5 ± 44.6	418.0	17.5	398.0	276.5	121.5	66.1	38.0	62.5	146.0	0.0	68.0
	F	Low-caffeine	137.0 ± 26.7	422.0	13.5	399.0	298.0	101.0	70.6	29.0	65.0	151.5	0.0	81.5

6	97	Standard	115.6 ± 30.1	642.5	46.0	517.5	489.5	28.0	76.2	84.5	193.5	189.5	0.0	106.5
	F	Low-caffeine	121.6 ± 22.4	630.5	108.0	402.5	266.0	136.5	42.2	120.0	9.5	232.5	0.0	24.0

7	95	Standard	120.1 ± 29.5	626.0	47.5	578.0	474.5	103.5	75.8	36.5	100.0	291.0	0.0	83.5
	F	Low-caffeine	128.6 ± 32.9	619.0	138.5	477.5	384.0	93.5	62.0	13.0	116.5	142.0	0.0	125.5

Mean ± SEM		Standard	145.1 ± 16.9	615.9 ± 34.3	39.2 ± 11.4	544.7 ± 37.4	422.3 ± 39.8	122.4 ± 38.3	68.3 ± 4.4	66.5 ± 15.9	112.4 ± 28.9	227.3 ± 43.4	0.1 ± 0	82.5 ± 12.3
		Low-caffeine	113.1 ± 11.3	615.1 ± 35.7	67.1 ± 19.7	524.9 ± 39.3	401.9 ± 41.9	123.1 ± 22.5	65.2 ± 4.9	39.9 ± 14.3	76.1 ± 15.9	240.2 ± 28.8	0 ± 0	85.6 ± 16.6

F, female; M, male; sAAm, salivary α-amylase in the morning; TIB, time in bed; SL, onset of sleep; SPT, total period of sleep; TST, Total sleep time; WASO, the time spent awake during the sleep period; SE, sleep efficiency; B2 WASO, the total awakening time during two hours before the final awakening; REM, rapid eye movement; N1, N2, light sleep; N3, slow-wave sleep.

## Abbreviations

Ala: Alanine
Arg: arginine
Asn: asparagine
Asp: aspartic acid
B2 WASO: early morning awakening
EEG: electroencephalogram
EGC: epigallocatechin
EGCG: epigallocatechin gallate
GABA: γ-amino butyric acid
Gln: glutamine
Glu: glutamic acid
HPLC: high performance liquid chromatography
N1/N2: non-REM sleep which is classified into light sleep (N1/N2)
N3: non-REM sleep which is classified into slow-wave sleep
PLG: polysomnography
REM sleep: rapid eye movement sleep
sAA: salivary α-amylase activity
sAAm: sAA in the morning
SE: sleep efficiency
Ser: serine
SL: onset of sleep
SPT: the total period of sleep
TIB: the time in bed
TST: total sleep time
VAS: visual analog scales
WASO: the time spent awake during the sleep period
